# Gauging the Impact of Gender Grammaticization in Different Languages: Application of a Linguistic-Visual Paradigm

**DOI:** 10.3389/fpsyg.2016.00140

**Published:** 2016-02-23

**Authors:** Sayaka Sato, Pascal M. Gygax, Ute Gabriel

**Affiliations:** ^1^Department of Psychology, University of FribourgFribourg, Switzerland; ^2^Department of Linguistics and English Language, Lancaster UniversityLancaster, UK; ^3^Department of Psychology, Norwegian University of Science and TechnologyTrondheim, Norway

**Keywords:** gender representation, gender stereotypes, grammatical gender, generic masculine, thinking-for-speaking hypothesis, bilingualism

## Abstract

Employing a linguistic-visual paradigm, we investigated whether the grammaticization of gender information impacts readers’ gender representations. French and German were taken as comparative languages, taking into account the male gender bias associated to both languages, as well as the comparative gender biases associated to their plural determiners (French: *les* [generic] vs. German: *die* [morphologically feminine]). Bilingual speakers of French and German had to judge whether a pair of facial images representing *two men* or *a man and a woman* could represent a gender stereotypical role noun prime (e.g., *nurses*). The prime was presented in the masculine plural form with or without a plural determiner. Results indicated that the overt grammaticization of the male gender in the masculine form dominated the representation of the role nouns (though interpretable as *generic*). However, the effect of the determiner was not found, indicating that only gender information associated to a human reference role noun had impacted readers’ representations. The results, discussed in the framework of the *thinking-for-speaking* hypothesis, demonstrated that linguistic-visual paradigms are well-suited to gauge the impact of both stereotype information and grammaticization when processing role nouns.

## Introduction

The ways in which languages organize specific concepts in their linguistic systems have been found to impact how we represent information (e.g., Gennari et al., 2002; Papafragou et al., 2002). This notion, further developed as the *thinking-for-speaking* hypothesis by Slobin (1996) in his work on motion events, proposes that the encoding of concepts and events within a language acts both as a foundational and constraining structure for how verbal information is represented. Processing a specific language therefore imposes speakers to focus on particular concepts that are grammaticized within its structure, resulting in language-bound representations. As will be further discussed in this paper, bilinguals are particularly suited for testing the thinking-for-speaking hypothesis as they offer a platform to examine the extent to which comprehension mechanisms change as a function of the characteristics of the language being used (e.g., Boroditsky et al., 2003; Fausey et al., 2010; Bylund and Jarvis, 2011). In the present study, we focus specifically on the case of gender representation during language comprehension, and argue that processing languages that grammaticize gender information in their linguistic structure will result in heightened biased representations of gender.

Recent psycholinguistic research investigating gender representation during language comprehension has shown that the presence or the lack of gender information in the linguistic structure of a language contributes to shaping distinct gender representations. For example, languages such as English, that do not systematically grammaticize gender information in their linguistic structure, encourage readers to rely on their world knowledge for gender representations (e.g., Carreiras et al., 1996; Kennison and Trofe, 2003; Oakhill et al., 2005; Reynolds et al., 2006; Pyykkönen et al., 2010). Reading about person references such as *nurse* will generate inferences about the possible gender of the depicted person, with gender stereotypes acting as a primary source for representation (e.g., Banaji and Hardin, 1996; Carreiras et al., 1996; Kennison and Trofe, 2003; Cacciari and Padovani, 2007; Kreiner et al., 2008). Banaji and Hardin (1996), for example, showed that participants’ judgments to the target stimuli (Experiment 1: judge whether the target was a male or female: *he vs. hers*; Experiment 2: judge whether the target was a pronoun or not: *she vs. do*) following either a gender stereotypical (e.g., nurse, mechanic) or gender definitional (e.g., mother, king) prime was found to be responded to faster when there was a gender congruency between the prime and target stimuli. Oakhill et al. (2005) further substantiated these effects of gender priming with a series of lexical priming experiments. Participants in their study were faster to accept word pairs consisting of a stereotypical role noun (e.g., surgeon) and kinship term (e.g., brother, sister) as referring to the same person in cases when the words were gender congruent. Activating such stereotyped gender inferences has been found to be immediate and robust among English readers, demonstrating that such role nouns may prime a specific stereotypical gender even if morphological or grammatical information may not compel readers to do so (e.g., Carreiras et al., 1996; Kennison and Trofe, 2003).

These representation tendencies, however, are not readily generalizable for readers of grammatical gender languages such as French or German, where stereotypical gender is only one of the two possible sources contributing to the construction of gender representations. In these languages, gender is also integrated as part of their grammatical structure. Grammatical gender thus classifies a specific gender category to all nouns (e.g., masculine, feminine, and neuter in the case of German). This gender feature, when marked on person references, commonly corresponds to the biological gender of the referent (i.e., masculine = man, feminine = woman)^1^, constraining its language users to consistently monitor gender information at both grammatical and semantic levels. A fundamental claim made by researchers is that the interaction between these two sources of information (i.e., stereotypical and grammatical) during the processing of role nouns is complex, and that the mechanisms for representing gender information are not always straightforward (e.g., Irmen, 2007; Garnham et al., 2012; Gygax et al., 2012; Esaulova et al., 2013).

The complexity of this interaction is rendered by the fact that gender information associated to its surface form does not necessarily coincide with its intended semantic connotations. For instance, when considering the masculine form, there is a discrepancy between form and meaning. Whereas role nouns such as *infirmières*_Feminine_ [nurses] marked in the feminine grammatical form refer unambiguously to female nurses, the masculine form (*infirmiers*_Masculine_) can refer exclusively to men (i.e., only male nurses) or it may refer to a group composed of both male and female persons (i.e., *generic* interpretation). Readers are presented with a challenge to disambiguate the intended interpretation of the masculine form. It has been argued that its surface forms naturally emphasize the association to the male gender, inevitably prompting a male-specific interpretation (e.g., Gygax et al., 2012). Gygax et al. (2012), for example, adapting Oakhill et al.’s (2005) paradigm in French, found that when participants were instructed to decide whether the person represented by a kinship term in pairs such as *tante* [aunt] – *infirmiers*_Masculine_ [nurses] could belong to a group represented by the second noun (always in the grammatical masculine plural form), they responded positively more often and faster when the kinship term was a man, indicating a male dominant representation. The authors concluded that the generic interpretation could only be activated through active processes, yet the male-specific interpretation was always passively activated (i.e., without control). Most studies using on-line (e.g., Gabriel and Gygax, 2008; Gygax et al., 2008) and off-line (Stahlberg et al., 2001; Braun et al., 2005) tasks concur on the male-specific impact of the masculine form. Crucially, however, this male bias effect persisted even when gender stereotypicality violated the grammatical gender information (as seen in *infirmiers*: female stereotype, masculine grammar), leaving the effect of stereotype information unclear.

In German, additional grammatical cues associated to its plural determiner (*die* [the]) and pronoun (*sie* [they]) have been investigated, especially in conjunction with possible female biases. In a study investigating gender representation in German, Rothermund (1998) found an unexpected reduction of the male bias when participants conducted a recognition task after reading texts including plural masculine references (*die*_plural_ *Studenten* [the students]). The male-attenuating effect was attributed as being triggered by the plural determiner *die* which shares the same surface form as the singular feminine determiner *die* [*the* – singular – feminine]. Garnham et al. (2012) also showed a male attenuated effect (or an additive female effect) when presenting the German plural pronoun *sie* (i.e., *they –* also feminine-equivalent) in a sentence judgment task examining the interpretations of masculine role nouns. When the same was done in French, however, the masculine pronoun *ils* [they – masculine *specific* or *generic*] did not have a male amplifying effect despite its male association. The authors argued that although cumulating grammatical cues do not augment male biases, combinations of male and female-equivalent grammatical cues may distract readers from activating male specific representations. To our knowledge, when looking strictly at determiners, only one study (e.g., Gygax et al., 2008) has generated specific hypotheses as to the impact of the definite plural determiner *die* in German, yet its female-bias effect (as shown by Rothermund, 1998) was never clearly replicated.

The studies discussed here demonstrate how grammaticized information influences readers’ comprehension processes. Grammatical gender languages work in a top–down manner, constraining their users to consistently monitor gender both on grammatical and semantic levels. If, as suggested by the thinking-for-speaking hypothesis, information grammaticized in languages prompts readers’ gender biases, which in turn anchor their representations, these regularities should also become evident on their representations. If this were the case, it is reasonable to assume that readers of more than one language may switch representations as they change languages. This notion is further developed in this study by looking particularly at bilinguals where the language biases of each of the bilingual’s languages should surface on their representations. Sato et al. (2013) followed this line of logic and investigated in a sentence-based paradigm, whether English-French bilinguals would construct different representations according to their first (L1) and second (L2) language. They presented English and French bilingual participants with sentence primes including role nouns with stereotypical gender (e.g., female: *nurses*, male: *politicians*, neutral: *pedestrians*). Participants judged the plausibility of target sentences including a gender reference (e.g., *some men, some women*) to be a sensible continuation of the prime. The results indicated that switching language was also accompanied by changes of biases in mental representations of gender, with English eliciting stereotyped representations and French male-biased representations triggered by the masculine form. Importantly, participants’ L2 proficiency was found to be a good indicator of the extent of the representation switch between L1 and L2.

In the present study, we followed Sato et al.’s (2013) study and investigated the effects of stereotypes and linguistic encodings of gender on the representation of person reference role nouns. French and German were taken as comparative languages, provided that they were both marked with grammatical gender. This made them ideal candidates to test thinking-for-speaking effects, as opposed to English, which lacks systematic grammaticization of gender. Characteristics surfacing on representations when processing French and German should essentially reflect the impact of how linguistic encoding contributes in shaping gender representations. Additionally, despite their common usage of the masculine form to denote a generic interpretation, gender associations linked to the plural determiners differ in the two languages. As argued by Rothermund (1998) and Gygax et al. (2008), the German determiner *die* [the – plural] shares the same surface structure as the singular feminine determiner *die* [the – singular – feminine], and should contribute to a female additive bias when presented with a role noun in the masculine form. In contrast, the French plural determiner *les* [the – plural] corresponds to both feminine and masculine nouns as they have a single morphological realization (i.e., gender syncretism: Corbett, 1991) and therefore should not enhance any additional gender information. If in the present study we are able to observe differences in gender biases between French and German representations, it should provide more compelling evidence as to the impact the grammaticization of language has on our conceptualization of gender information.

To test these effects, we employed a combined linguistic-visual paradigm. This paradigm was intended to provide a more sensible experimental framework to address the immediacy of gender activation. While a handful of studies have examined gender representation processes employing a lexical-based paradigm (Banaji and Hardin, 1996; Oakhill et al., 2005; Cacciari and Padovani, 2007; Gygax et al., 2012; Siyanova-Chanturia et al., 2012), none have directly addressed the impact of the use of the masculine form, or of role noun determiners. Studies investigating these effects have approached the issue with a sentence comprehension task, applying anaphor resolution paradigms that were dependent on the detection of semantic and syntactic inconsistencies in comprehension. These tasks therefore did not strictly speak to the immediacy of the activation of such surface-level grammatical cues, and discursive contextual elements may have interfered with stereotype activation or with the accessing of signals during activation. More importantly, although moderate, some effects of stereotype have been observed, indicating that teasing apart these effects in a linguistic context has been complex.

For instance, Esaulova et al. (2013) found a subtle effect of stereotypical gender in German. In their experiment (Experiment 1), participants were presented with sentences composed of an anaphor (e.g., *er* [he]) and a stereotyped role noun (e.g., *der Elektriker* [the electrician]) as an antecedent while their eye movements were recorded. Although comprehension difficulty was most prominent when the anaphor did not agree grammatically with its antecedent, as illustrated by most eye-tracking measures, sentence processing was also influenced by the role nouns’ stereotypicality, as demonstrated in the late measures only (e.g., regression path on the pronoun region and total fixation path on the role noun). Following the aforementioned Banaji and Hardin’s (1996) experiments, Cacciari and Padovani (2007) and Siyanova-Chanturia et al. (2012) also reported stereotype effects in Italian, a grammatical gender language. They found that when a pronoun (e.g., *lui* [he] or *lei* [she]) was primed by a bi-gender role noun (a noun that can vary in grammatical gender as a function of biological gender, as in *insegnante* [a female/male teacher]), participants were particularly slow to decide whether the pronoun was masculine or feminine when primed by a counter-stereotypical role noun. Additionally, Carreiras et al. (1996), in a self paced reading task (Experiment 2), showed that Spanish participants reading was delayed when a role noun (e.g., the carpenter) was written in a grammatical form that mismatched its stereotypicality (e.g., *La carpintera_Feminine_* [the female carpenter] or *El enfermero_Masculine_* [the male nurse]).

In sum, most studies have shown a strong impact of grammatical gender, with some authors claiming that grammatical gender had only overshadowed stereotype effects (e.g., Irmen, 2007; Esaulova et al., 2013; Reali et al., 2015). Although, the impact of grammatical cues seems central in representation processes, the reasons for the overriding effects of grammatical cues over gender representations have not been clearly shown. We therefore explore the possibility that the prevalence of male representations in grammatical gender languages (and the lack of stereotype effects) may have well been prompted by the very nature of the paradigms being employed, provided that both the prime and target stimuli were verbal stimuli. The use of verbal target stimuli, maintaining a close link with its verbal prime, may have resulted in mental representations that reflected only and merely linguistic activations. It could be that processing both prime and target stimuli in a verbal context may constrain readers to over-monitor grammatical and syntactical properties. This monitoring in turn may enhance the signal of a representation based on linguistic cues (i.e., toward a male bias in gender-marked languages). In contrast, linguistic-visual paradigms have been found to be effective in gauging effects of gender priming. Studies in social psychology have shown that gender priming may be observed by presenting gender associated words (e.g., Kawakami and Dovidio, 2001: stereotypical traits; Lemm et al., 2005: words with gender-specific suffixes and role nouns) followed by picture targets that required participants’ judgments. For instance, Lemm et al. (2005) showed that although past studies indicated a weaker priming effect when using cross-modal paradigms, the gender priming effects found in their study were still large. Consequently, this approach may indeed be well-suited to gauge the subtle stereotype effects we seek to explore.

In our task, stereotypical role nouns in the masculine plural form, either with or without a plural determiner, served as gender primes in German or in French. Participants had to make judgments as to whether a visually presented pair of faces (male pairs or mixed pairs of faces composed of a woman and a man) that followed could represent the preceding prime. The composition of face pairs represented the possible interpretations that the role noun in the masculine form holds (i.e., a male specific or a generic interpretation). We expected to replicate the male bias demonstrated in previous findings (i.e., facilitated responses to male pairs of faces), and intended to explore the influence of stereotype information. Specifically, an attenuated male bias was expected in the female and possibly the neutral stereotyped conditions. Importantly, we also expected that the determiner *die* in German would attenuate this potential male bias arising from the masculine form of the role noun, whereas French rolenouns would retain the male bias. Finally, the experimental task was carried out in participants’ L1 and L2 to examine any representational shift that would be prompted by the regularities of each language. For participants’ L2, we also took L2 proficiency into account, as measured by a L2 C-test. We expected shifts of representations to be influenced by L2 proficiency (as in Sato et al., 2013).

## Materials and Methods

### Participants

#### Native German Group

Fifty Caucasian German-speaking students from the University of Fribourg (Switzerland) participated in the experiment for course credits. All participants were native speakers of German whose L2 was French (mean age: 22, mean start age of French acquisition: 9.4 years, mean number of schooling of French as L2: 7.2 years). Forty-one participants were women^2^.

#### Native French Group

Fifty-one Caucasian French-speaking students from the University of Fribourg participated in the experiment for course credits. All participants were native speakers of French whose L2 was German (mean age: 22, mean start age of German acquisition: 7.5 years, mean number of schooling of German as L2: 9.2 years). Thirty-nine participants were women.

### Materials

#### Prime Role Nouns

Thirty-six gender stereotypical role nouns were selected as primes for the experiment (see **Table 1**). These role nouns were taken from Gygax et al. (2008), all of which were normed and tested for gender stereotypicality in Gabriel et al. (2008) in both German and French. Role nouns were female (e.g., nurses [*Krankenpfleger*/*infirmiers*]), male (e.g., bosses [*Arbeitgeber*/*patrons*]) or neutral (e.g., pedestrians [*Spaziergänger*/*promeneurs*]) in stereotype. To ensure that both female and male stereotyped role nouns were similarly judged as prototypical exemplars of their respective stereotype, we inverted ratings to female stereotypes (i.e., new rating = 100 – initial ratings), and conducted a *t*-test to ensure that both were similarly judged. As expected, both were similar in both languages, *t_French_*(22) = 0.23, *p* = 0.82, and *t_German_*(22) = 0.47, *p* = 0.64.

**Table 1:** Role nouns from Gabriel et al. (2008) and their corresponding gender proportion and standard deviations (in parentheses) for each stereotype.

English	German	% (*SD*)	French	% (*SD*)
Spies	Spione	67 (15)	Espions	74 (17)
Golfers	Golfspieler	68 (14)	Golfeurs	73 (16)
Politicians	Politiker	69 (11)	Politiciens	72 (13)
Police officers	Polizisten	69 (10)	Policiers	70 (13)
Statisticians	Statistiker	72 (12)	Statisticiens	74 (15)
Bosses	Arbeitgeber	72 (12)	Patrons	74 (16)
Computer specialists	Informatiker	79 (11)	Informaticiens	67 (22)
Surgeons	Chirurgen	75 (12)	Chirurgiens	75 (14)
Technicians	Techniker	78 (14)	Techniciens	75 (14)
Engineers	Ingenieure	78 (11)	Ingénieurs	74 (14)
Physics students	Physikstudenten	81 (11)	Etudiants en physique	67 (28)
Pilots	Flieger	76 (13)	Aviateurs	74 (17)

***Mean***		74 (5)		72 (3)

Singers	Sänger	45 (8)	Chanteurs	48 (9)
Pedestrians	Spaziergänger	46 (8)	Promeneurs	52 (13)
Cinema goers	Kinobesucher	49 (6)	Spectateurs de cinéma	50 (5)
Concertgoers	Konzert-Zuhörer	47 (7)	Auditeurs de concert	51 (10)
Schoolchildren	Schüler	48 (5)	Ecoliers	53 (13)
Spectators	Zuschauer	41 (8)	Spectateurs	51 (5)
Neighbours	Nachbarn	50 (5)	Voisins	50 (8)
Swimmers	Schwimmer	50 (9)	Nageurs	50 (10)
Tennis players	Tennisspieler	52 (7)	Joueurs de tennis	54 (8)
Authors	Autoren	52 (9)	Auteurs	54 (8)
Musicians	Musiker	50 (9)	Musiciens	59 (13)
Skiers	Skifahrer	53 (8)	Skieurs	55 (9)

***Mean***		49 (3)		52 (3)

Beauticians	Kosmetiker	11 (8)	Esthéticiens	18 (20)
Birth attendants	Geburtshelfer	11 (19)	Assistants maternels	18 (18)
Fortune tellers	Wahrsager	24 (16)	Diseurs de bonne aventure	28 (27)
Cashiers	Kassierer	27 (16)	Caissiers	24 (15)
Nurses	Krankenpfleger	24 (11)	Infirmiers	30 (11)
Hairdressers	Coiffeure	21 (11)	Coiffeurs	38 (25)
Psychology students	Psychologiestudenten	25 (29)	Etudiants en psychologie	33 (10)
Dieticians	Diätberater	27 (15)	Diététiciens	37 (22)
Dressmakers	Schneider/Näher	23 (13)	Couturiers	40 (32)
Dancers	Tänzer	33 (12)	Danseurs	29 (14)
Sales assistants	Verkäufer	33 (14)	Vendeurs	37 (13)
Social workers	Sozialarbeiter	41 (14)	Assistants sociaux	33 (15)

***Mean***		25 (9)		30 (7)


*All role nouns are presented in the plural form as was in the experiment.*

#### Target Face Pairs

The face pairs were created with the face modeling software FaceGen Modeler program version 3.1.4 (Singular Inversions Inc, 2004; Toronto). A total of 30 male and 30 female Caucasian faces with neutral expressions were created. They all had neutral expressions and the crown area of the faces were removed in order to eliminate possible biases associated with certain hairstyles evoking gender-biased information.

Twenty-one participants (14 women and seven men who did not participate in the main experiment) participated in the first norming phase by rating the gender typicality of all 60 faces on a 7-point scale (very masculine = 1, very feminine = 7) on a paper–pencil administrated questionnaire. Presentation order of the faces was randomized for each participant. Only faces that were clearly rated as female (i.e., average score > 5) or male (i.e., average score < 3) were selected for the experiment. Twenty-four female faces (*M* = 5.72, *SD* = 0.33, range = 5.43–6.3) and all thirty male faces (*M* = 1.58, *SD* = 0.26, range: 1.23–2.47) were retained. The average ratings of the female faces [*t*(23) = 25.27, *p* < 0.001; *M_difference_* = 1.72] and male faces [*t*(29) = -50.17, *p* < 0.001; *M_difference_* = -2.42] were significantly different from the scale midpoint (i.e., 4), with the difference being bigger for male faces than for female faces. We deemed this imbalance in deviation from midpoint non-problematic for the purpose of our study, as our main focus was on assuring to select non-ambiguous faces.

**FIGURE 1:**
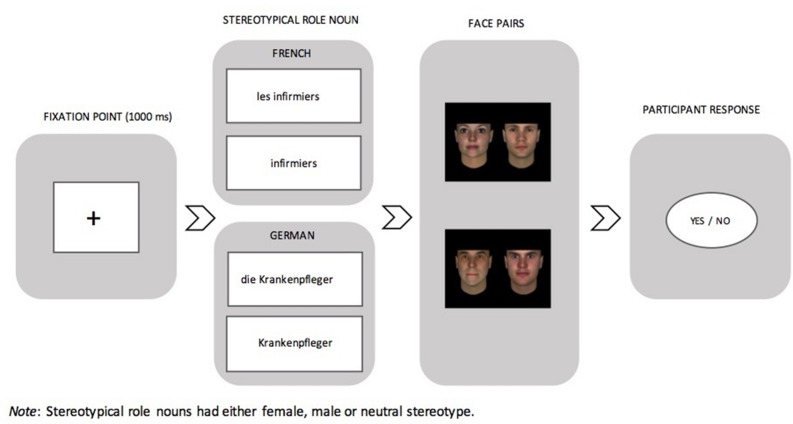
**The experimental procedure in each condition**.

The 54 faces were then combined to make *male* and *mixed pairs of faces* (see **Figure 1** for an example of a presented pair of faces). Female pairs of faces were not constructed for the experiment, as the interpretation of the presented masculine forms could not be grammatically interpreted as being female-specific (i.e., represented by female pairs of faces). More importantly, these female pairs of faces were avoided based on findings by Gygax and Gabriel (2008) who demonstrated that the presentation of both feminine and masculine forms in the same experiment directs readers toward a stronger male-specific representation of the masculine form. Female faces for mixed pairs were always presented on the left in order to avoid a male preferred response according to a possible left-side bias, illustrated in past studies using response scales in left-to-right languages (e.g., Gabriel et al., 2008). All pairs of faces were comprised of different faces.

A second norming phase was conducted in order to ensure that male and mixed pairs of faces were not processed differently due to perceptual properties that we had not foreseen. In this pilot experiment, our experimental pictures were presented on a computer screen running Experiment Builder (SR Research) to another group of 27 participants (25 women and six men who had not participated in the first norming phase). Their task was to decide, on two blocks of trials, whether the presented pairs of faces were *of the same sex* in one block or *of different sex* in the other block, by indicating their responses with a *yes* or *no* button press. The block order was inversed for half of the participants. A repeated-measure ANOVA on correct response times (i.e., 94% of the data) showed no main effect of *block*, *F*(1,26) < 1, *ns.*, no main effect of *faces*, *F*(1,26) = 3.18^3^, *ns.*, and no interaction, *F*(1,26) = 1.75, *ns*., confirming the homogeneity of our experimental target stimuli in terms of perceptual properties.

#### L2 Proficiency Assessment

Participants’ L2 proficiency levels were operationalized by their performance scores on a given C-test (as done in Sato et al., 2013). Commonly in a C-test, participants are given several distinct passages in which the second half of every other word is deleted except for the first and last sentences. The task is to restore the blanks in the allocated time. This procedure was developed as an effective measurement substituting cloze tests that were used in earlier years, and in recent years, has been frequently encouraged as a measure for language proficiency (Grotjahn et al., 2002; Eckes and Grotjahn, 2006).

In fact, C-tests have been shown to be highly correlated with standardized tests (e.g., Studienkollegs in German: Grotjahn and Allner, 1996; TOEFL in English: Hastings, 2002; the five competencies of the Test de Connissance du Français: Reichert et al., 2010). We employed the German C-test offered by onDaF^4^ to test German proficiency. Score ratings on this test are considered equivalent to the Common European Framework of Reference for the levels A2 to C1. French proficiency was evaluated with Coleman’s (1994) C-test. Four texts were chosen from each original version and 20 min were allocated to complete the task.

#### Role Noun Translation Task

To verify whether participants correctly identified the role nouns presented in L2, a role noun translation task was conducted after the experimental trials. Participants were asked to provide a translation for each presented role noun in their L1.

### Design and Procedure

The experimental task was conducted first in L1, followed by the task in L2 to minimize any data contamination during the processing of a less dominant language^5^. Two experimental lists were created to ensure that a role noun would not appear in both languages for a given participant. The two lists were symmetrically different, in that if a role noun appeared in French in List 1, in List 2, it would appear in German. To avoid an imbalance of gender stereotypicality between languages, role nouns of similar strength of stereotype were always allocated to each language (see **Table 1**). Each list consisted of six female, six male and six neutral role nouns per language, resulting in 36 critical role nouns per list, with each role noun appearing only in either French or German. Each role noun was presented four times per participant (Oakhill et al., 2005; cf. Gygax et al., 2012, for a similar procedure): twice with a determiner (once followed by male pairs, once by mixed pairs of faces), and twice without. All experimental items were intended to elicit a *yes* response.

To trigger *no* responses, twenty filler role nouns that had a gender association by definition (e.g., grandmother: *Großmütter*/*grand-mères*) were included. Half of the filler role nouns were male by definition, whereas the other half were female. These filler primes were also presented four times with their respective determiner allocations and face pairs. As these nouns were not ambiguous in terms of gender, including them prevented participants from responding *yes* throughout the experimental task without truly processing the role nouns and the target stimuli.

The study was accepted by the Ethics Committee at the Department of Psychology of the University of Fribourg and conformed to relevant regulatory standards. All participants were granted informed consent. For each experimental trial, participants were first presented with a gender stereotypical role noun prime following a fixation point (1000 ms). The role noun was presented in the masculine plural form either in conjunction with a plural definite determiner (e.g., *die Ingenieure/les ingénieurs* [the engineers]) or without (e.g., *Ingenieure/ingénieurs* [engineers]). Participants were instructed to press the *yes* button after having read the presented role noun, which prompted the presentation of a picture of a pair of faces. Their task was to judge as quickly as possible with a *yes*/*no* button press whether the presented target face pairs could represent the prime role noun that appeared prior to the faces (see **Figure 1** for the procedure). Filler trials, which were randomized among experimental trials followed the same procedure, and the role nouns within them were also presented either with or without a determiner.

The experiment was run on a Power Macintosh 4400 with the Psyscope software (Cohen et al., 1993) connected to a button box to provide millisecond accuracy responses. Two buttons were labeled, one “*Ja*” (yes) and the other “*Nein*” (*no*) for German-speaking participants and “*Oui*” (yes) and “*Non*” (no) for French-speaking participants. Items were presented on a computer screen and the “*Ja*/*Oui*” button was always pressed by the participant’s dominant hand. All participants were individually tested in a quiet room, with instructions being given in their respective native languages. They underwent a practice session in their L1 with four items to familiarize themselves with the task and procedure.

After the main experimental task, three paper-based post-tests were conducted. First, participants were given a C-test in their respective L2. Following the C-test, participants were requested to assess their L2 competence in terms of their listening, reading, writing and speaking abilities in the L2 and to indicate the years and age of L2 acquisition by means of a self-administered questionnaire. Finally, the role noun translation task was given to the participants to ensure they had properly processed the critical items.

## Results

We conducted analyses on both participants’ binary responses (*yes/no*) to the facial images and their response times for yes-responses (i.e., accepting the faces). Based on the results of the role noun translation task conducted after the main experimental task, items in the L2 that were frequently unknown to each language group (fewer than 10% of the participants were able to provide a correct translation) were omitted from the analyses (*Schneider* [dress makers] and *Wahrsager* [fortune tellers] were removed from L2-French participants’ data and *diseurs de bonne aventure* [fortune tellers] from L2-German participants’ data). Mixed-effects logistic regression was used to model the binary outcome variable (yes/no responses), and linear mixed-effects regression was used to model participants’ positive response times. Mixed-effect models provide a means to perform analyses that account for missing values and to avoid the language-as-a-fixed-effect fallacy (Brysbaert, 2007). All analyses were conducted using the R software (R Core Team, 2013), with the *glmer* and *lmer* function from the *lme4* package (Bates et al., 2014). As suggested by Barr et al. (2013) a model with a maximal random factor structure was adopted. Random intercepts and slopes were varied for participants and items in order to account for the variance in performance created by the factors (Baayen et al., 2008). Random slopes were eliminated if their removal did not result in a significant amelioration of the model or if the model did not converge. All predictors for fixed effects were sum coded (+1, -1) and were entered by step-wise forward selection to an initial null model. Given that participants’ L2 *proficiency* was expected to predict general performance in the L2, the *proficiency* predictor, as measured by C-test scores, was centered and entered as a covariate in the null model, which included only random effects. Analyses for each language group were conducted separately as in Sato et al. (2013), given that we expected different variances in the C-test scores. Indeed, C-test difficulty has been found to vary according to various factors such as the language of the C-test, text type or deletion pattern (Sigott and Köberl, 1996).

Log-likelihood ratio tests were used to determine the adequacy of including each predictor in the model. A more complex model including the predictor in question was compared to a simpler model without the inclusion of the predictor. If its integration significantly improved the model, the predictor was retained within the model. The predictors tested in the models were *face pairs* (male vs. mixed pairs of faces), *stereotype* (female vs. male vs. neutral), *task language* (German vs. French) and *determiner* (without determiner vs. with determiner).

### Responses to Facial Targets

Participants’ binary choices were modeled in a mixed logit model to predict the likelihood that participants would accept a face pair presented to them after a particular role noun prime. For both language groups, the first model that followed the null model tested the effects of the masculine form and of stereotype by introducing *face pairs, stereotype* and their interaction to the null model. For both groups, the inclusion of these predictors significantly improved the model fit (Native German group: χ^2^ = 205.36, *df* = 5, *p <* 0.001; Native French group: χ^2^ = 150.8, *df* = 9, *p* < 0.001). The second model proceeded to test whether the effect of the German determiner impacted the interpretation of the presented prime by adding the main effects of *task language* and *determiner*, and importantly, all interactions between *face pair, task language* and *determiner*. While this lead to a significant improvement of the model for the native German group (χ^2^ = 75.8, *df* = 6, *p <* 0.001), the model failed to converge for the native French group. Therefore, only main effects for *task language* and *determiner*, as well as the interaction between *task language* and *face pairs* were introduced into the model, indicating an improvement (χ^2^ = 31.69, *df* = 5, *p <* 0.001). As for the random structure, the final model for the native German group included random slopes for *determiner* at the item level. The model for the native French group included random slopes for *face pair* at the item level and *stereotype* and *task language* at the participant level. Both models indicated a variance inflation factor less than 1.5, indicating that collinearity was not an issue.

#### Native German Group

**FIGURE 2:**
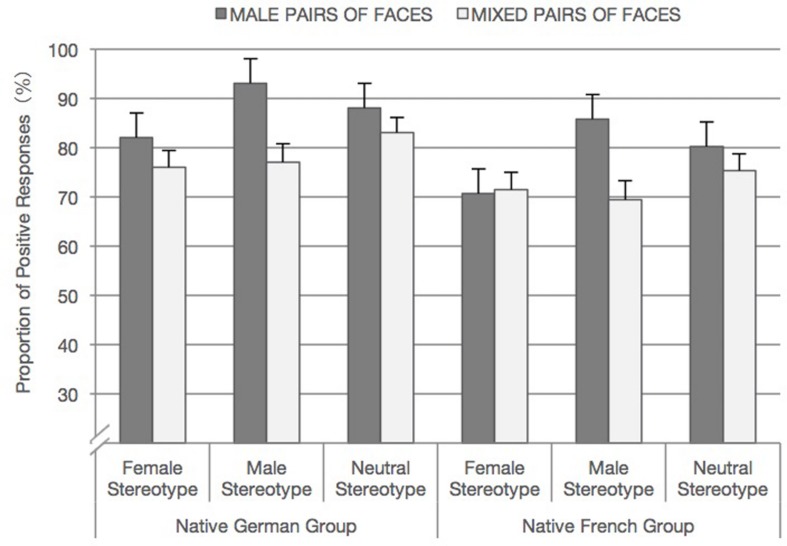
**Mean proportion of positive responses to accept facial images for each native group in each stereotype condition (independent of *task language*).** Error bars indicate standard errors.

The results showed significant main effects of *face pairs* and *stereotype* which were qualified by a significant interaction. Overall, the likelihood of a positive response was substantially higher for male pairs of faces than for mixed pairs of faces (*b* = 0.47, *SE* = 0.04, *p <* 0.001, odds ratio = 2.56). Consistent with our predictions, the *face pairs* X *stereotype* interaction revealed that this preference for male pairs of faces was especially pronounced when they followed role nouns with male stereotype, compared to when they followed role nouns with neutral stereotype (*b* = 0.43, *SE* = 0.06, *p* < 0.001, odds ratio = 2.36) or female stereotyped role nouns (*b* = 0.68, *SE* = 0.2, *p <* 0.001, odds ratio: 3.89; see **Figure 2**).

The model also revealed main effects of *determiner* and *task language*, indicating that the likelihood of receiving positive responses was higher if face pairs were preceded by role nouns with an article than when presented without the article (*b* = -0.19, *SE* = 0.05, *p* < 0.001, odds ratio = 0.68). Face pairs were also more likely to be responded to positively when they were presented with role nouns in participants’ L2 French than when presented with role nouns in their dominant L1 German (*b* = -0.09, *SE* = 0.04, *p <* 0.05, odds ratio = 0.83). Contrary to our predictions, these two predictors did not interact, which would have supported the effect of the German determiner *die*. However, a *face pairs* by *task language* interaction surfaced, indicating that male pairs of faces were more likely to be responded to positively when preceded by a role noun in participants’ L1 German than when preceded by a role noun in their L2 French (*b* = 0.24, *SE* = 0.04, *p <* 0.001, odds ratio = 1.62).

#### Native French Group

As was the case for the German group’s responses, the analysis of the French sample revealed a significant main effect of *face pairs* and a marginal significant effect of stereotype further qualified by their significant interaction. The likelihood of accepting face pairs was again higher for male pairs of faces than for mixed pairs of faces (*b* = 0.31, *SE* = 0.05, *p* < 0.001, odds ratio = 1.86). The interaction revealed that the likelihood for participants to accept men pairs of faces was again substantial when they followed male stereotyped role nouns than when they followed neutral (*b* = 0.42, *SE* = 0.07, *p <* 0.001, *p <* 0.01, odds ratio = 2.31) or female stereotyped role nouns (*b* = 0.75, *SE* = 0.12, *p <* 0.001, odds ratio = 4.48; see **Figure 2**).

While the model revealed a main effect of *determiner* indicating that role nouns without a determiner triggered greater positive responses (*b* = 0.08, *SE* = 0.03, *p <* 0.05, odds ratio: 1.17), no interactions involving this predictor were observed. Finally, as was the case for the native German group, a significant *face pairs* by *task language* interaction indicated that responses to accept men pairs of faces was greater in the dominant L1 French than in the L2 French (*b* = -0.08, *SE* = 0.03, *p <* 0.05, odds ratio = 0.85).

### Positive Response Times to Facial Targets

Overall, both groups responded above chance level to accept facial targets (native German group: *yes* responses = 83%, *no* responses = 17%; native French group: *yes* responses = 75%, *no* responses = 25%), although these items were intended to elicit positive responses. Only reaction times to these positive responses were subject to analyses. Response times that were 2.5 standard deviations above or below the participant’s mean were replaced by their cut-off values (3.5%). Following the analyses of participants’ responses to face pair targets, the effects of the masculine form and stereotype were examined by introducing the main effects of *face pairs, stereotype* and their interaction to the null model. There were significant improvements to the models for each language group (Native German group: χ^2^ = 157.67, *df* = 5, *p <* 0.001; Native French group: χ^2^ = 210.94, *df* = 5, *p <* 0.001). The second model then added the main effects of *task language* and *determiner* and all interactions between *face pair, task language* and *determiner* in order to test the impact of the German determiner. The additions of these predictors resulted in an improvement of the models for the native German group (χ^2^ = 123.25, *df* = 6, *p <* 0.001) but not for the native French group. Given that none of the effects introduced in the second model were significant, the initial model was retained as the final model. For the native German group, the random structure for the final model included random slopes for *face pairs*, *determiner* and *task language* at the item level and *face pairs*, *stereotype*, *task language* and *determiner* on the participant level. The model for the native French group included random slopes for item level and *face pairs, stereotypes* and their interaction for participant level. Collinearity was not an issue given that both models indicated a variance inflation factor less than 1.9.

#### Native German Group

Consistent with the analyses for participants’ responses, the final model showed significant main effects of *stereotype* and *face pairs*, which were qualified with their significant interaction. Male pairs of faces (825 ms) were responded to significantly faster than mixed pairs of faces (995 ms; *b* = -108.5, *SE* = 29.04, *t* = -3.73) confirming the male bias in past studies. This male bias was more prevalent when role nouns preceding facial targets were of male stereotype than when they were neutral (*b* = -47.64, *SE* = 13.89, *t* = -3.43) or female (*b* = -60.47, *SE* = 24.27, *t* = -2.49) stereotype. No main effects or interaction effects including *determiner* were found, but a significant *task language* effect indicated that participants were faster to respond in their L2 French (824 ms) than in their L1 German (991 ms).

#### Native French Group

The model revealed significant main effects of *stereotype* and *face pairs* that were further qualified by their significant interaction. Participants responded to male pairs (870 ms) of faces significantly faster than to mixed pairs (1009 ms) of faces (*b* = -75.83, *SE* = 15.88, *t* = -4.77) confirming a male bias. This effect was stronger for responses to male pairs of faces following male stereotyped role nouns than when following neutral (*b* = -52.71, *SE* = 13.62, *t* = -3.87) or female (*b* = -77.92, *SE* = 23.78, *t* = -3.28) stereotyped ones. Contrary to our initial expectations, no effects including *determiner* were significant.

## Discussion

The aims of the present study were twofold. First, we aimed to evaluate how linguistic encoding of gender in different languages shape and shift gender representations. Bilinguals of German and French were tested to assess the rather inconclusive effects of a female bias associated to the German determiner *die* (gender non-specific in the plural, but sharing the same surface form as the feminine singular determiner). Although the activation of a male bias was anticipated, the presence of an additional female association (i.e., *die*) was expected to attenuate this male bias in German. The second goal was to provide more compelling evidence of main and interaction effects when both stereotypical and grammatical gender information are available during the processing of role nouns. It has been argued that the impact of gender stereotype information has often been overshadowed by grammatical gender information in past studies, resulting in some uncertainty as to how stereotype information actually influences the interpretation of the masculine form. While past studies have relied on verbal targets, we argue that these tasks may have reinforced the grammatical and morphological cues being tested. Such an impact may have resulted in strong, yet less generalizable grammatical-based representations. In order to overcome these issues, a new experimental approach using visual targets was suggested.

Overall, we found a consistent main effect of *face pairs* for both of our groups, where responses to male pairs of faces were facilitated over mixed pairs of faces. This facilitation reflects the general ease in interpreting role nouns in the masculine form as being male-specific rather than generic. Although the surface form of the masculine grammar can theoretically be detached from its semantic association *masculine = men*, it nonetheless boosted the activation of semantic properties associated to the male gender. This was true even when participants were presented with visual targets. Importantly, this male bias was persistent despite the fact that our pilot experiment on the facial images showed a slightly faster, although not statistically significant (*p* = 0.08), tendency to process mixed pairs of faces. Our results therefore suggest that a strong male bias is indeed generated by the grammatical masculine form, and is not simply an artifact of the experimental tasks employed in previous studies.

However, for both language groups, participants’ responses to facial targets were also influenced by stereotypicality, with male stereotyped role nouns generating processing facilitation of following facial targets. In contrast, both response choices and positive response times indicated that facial targets following role nouns with a female stereotype were more difficult to process. We believe this to be indicative of an interference between the grammatically masculine form and the role noun’s female stereotypicality. Namely, both sources of information compete, increasing processing difficulty. In contrast, an advantage was observed (i.e., a greater likelihood of allocating positive responses and an elicitation of faster response times) for targets following male stereotyped role nouns, which suggests that the congruency between the grammatically masculine gender and stereotypical gender facilitated participants’ construction of mental representations.

Importantly, these main effects were further qualified by a consistent *stereotype* by *face pair* interaction for both the German and French group. This interaction indicated that participants’ acceptance to face pairs changed as a function of the stereotypicality of the role noun preceding it. Male stereotyped role nouns triggered the greatest facilitation to accept male pairs of faces. These results support the idea that when reading a gender associated role noun such as *nurses* (*Krankenpfleger*_German_, *infirmiers*_French_), or *bosses* (*Arbeitgeber*_German_, *patrons*_French_) in a grammatical gender language, gender stereotypical information is immediately activated as part of the information associated with the role noun. As we did not embed our primes within sentences, our results suggest that this activation is made at the lexical access, with discursive text elements not needed to guide the activation of gender stereotypical information. Although we did find evidence that the masculine form was highly influential in guiding the representation toward a male-dominant representation as found in previous studies, we also documented that readers rely on immediate stereotypical information, even in the presence of a masculine grammatical form.

Our results, however, do not necessarily speak to whether, and to what extent, grammatical gender or stereotypical information has a greater influence over gender representations, as discussed in some discourse-based studies (Irmen, 2007). They mainly support the idea that both are activated at an early stage (i.e., lexical access), a claim that contrasts those of anaphor resolution studies that suggest an activation at later stages of comprehension (e.g., Irmen, 2007; Esaulova et al., 2013). The absence or weak indications of immediate stereotype effects in past studies could be attributed to several reasons. First of all, past research has frequently relied on verbal primes *and* verbal targets (e.g., Gygax and Gabriel, 2008; Gygax et al., 2012) to substantiate a persistent effect of the masculine form as specifically referring to men, with the effects of stereotype being only modest. The present study, however, demonstrated that the apparent lack of stereotype effects could be attributed to the tasks used to investigate these issues. We believe that by using facial images as targets, we went beyond simple language-on-language task effects. Essentially the conceptual nature of stereotypes may have made them better candidates for non-verbal tasks which made it possible to delineate the true and noteworthy interaction between grammar and stereotypes when constructing a representation of gender. Another plausible argument for the absence of stereotype effects in past studies can be accredited to the nature of stereotype information, which dwindles rapidly as readers process discourse. Consequently, its effects did not clearly surface in previous studies on text comprehension. In the present study, the lexical-based paradigm may have allowed stereotype effects to surface before fading away, as they would have in a discursive context. Such a view may also support the reason for grammatical gender information to show a greater impact in most studies on the topic.

In terms of the impact of language shaping gender representations, the two language groups showed similar representation regularities in both their L1 and L2. This was rather unexpected given that we had anticipated the male bias to be reduced when participants processed the role nouns in German, due to its female-associated determiner. In fact, the German determiner did not elicit any substantial effects. Although there was a modest trend for mixed pairs of faces to be accepted more often when following female and neutral stereotyped role nouns (proportion of positive responses) when adding the determiner *die* for native German readers in L1, it did not lead to statistically significant effects. One could argue that when readers are faced with determining the grammatical gender of a noun, they will make use of available semantic (i.e., conceptual) and phonological information, which may result in processing facilitation (Schiller et al., 2003). In terms of our study, although both conceptual and masculine grammatical gender information competed to represent a probabilistic gender of the role noun, the association to the female gender of the German determiner did not substantially contribute in the representation process.

Although we cannot definitively refute the phenomenon, the male-attenuating effect in German documented by Rothermund (1998) appears to be at best superficial, at least in relation to the male-bias exerted by masculine forms. The fact that Garnham et al. (2012) found an effect of *sie* [they_Female_], was most likely due to the fact that they combined *die* and *sie*, both feminine equivalent, which offered a cumulative effect in deterring readers’ attention from the role nouns’ masculine form. In our German data, we observed only a main effect of *determiner*, whereby role nouns presented with a determiner facilitated responses to targets. These effects could be explained by the different rules associated to German. For instance, in French, although a noun must always be accompanied by a determiner even when a general statement is being made (e.g., *Infirmiers* doivent s’occuper des personnes. [*Nurses* need to care for people.] is grammatically incorrect: an article is always needed), in German, a noun can be presented both with and without a determiner (*Krankenpfleger* müssen sich um Menschen kümmern. [*Nurses* need to care for people.] vs. *Die* Krankenpfleger müssen sich um Menschen kümmern. [The nurses needed to care for people.]) which denote different meanings. The presence of *die* more clearly specifies that the role noun refers to *a* (particular) group of people, and not to the general activity represented by the role noun, consequently facilitating subsequent associated targets. In this regard, our German group may have constructed different representations according to whether the role noun was presented with or without a determiner.

We thus believe that gender information associated to the determiner appears to be trivial, at least in comparison to the information associated to the gender inflection on the role noun. This gender inflection might be particularly relevant with person reference nouns, as they integrate conceptual gender as part of their lexical representation (Oakhill et al., 2005). In contrast, information linked to a function word, such as a determiner that connote less content and semantic information, would be less readily associated to any conceptual gender. Nonetheless, these results are in line with the numerous studies suggesting that the male bias exerted in grammatical gender languages is strong and appears to govern the comprehension processes. As such, our results substantiate the idea that language contributes in guiding mental representations. In our study, the grammatical masculine form contributed in shaping male-dominant representations across (more or less) all stereotypes, which is at odds with the idea that the masculine is the *unmarked* gender in grammatical gender languages. Although the impact of *die* in German was not observed, the effects of the masculine form of the role nouns lend support to the idea that grammatical markings may well direct (or bias) our attention to particular categories. The masculine form makes the male concept more accessible to readers. Note that this bias may not extend to less ambiguous cases such as *bigender* nouns in Italian, as investigated, for example, by Cacciari and Padovani (2007) or Siyanova-Chanturia et al. (2012).

Interestingly, we also observed a *task language* by *face pair* interaction surfacing in our German group’s responses, suggesting that the male bias was more persistent in participants’ L1 German than in their L2 French. This is crucial given that their dominant language exerted a greater male bias than their less fluent L2, despite having a better understanding and command of the language and the different interpretations of the masculine form in their L1. These results hint that the male bias stem from L1 for grammatical gender language readers. Such an account is in line with bilingual processing theories proposing that the languages of a bilingual are non-selectively activated even when only one language is being used for language comprehension processes (Dijkstra and van Heuven, 1998; de Groot et al., 2000).

Finally, we highlight that our linguistic-visual paradigm served as an effective approach to gauge the effects in question. The male bias and stereotype effects observed in our study were apparent in both the participants’ L1 and in their less dominant L2. Importantly, despite the lack of stereotype effects observed in the presence of a strong masculine cue in past studies, our paradigm allowed us to observe stereotype effects. While some researchers argue that mixed-modal paradigms produce less priming effects (e.g., Alario et al., 2000), our studies concurred with the conclusions made by Lemm et al. (2005) that they are still very efficient and powerful.

Our results suggest that thinking to speak or read in a grammatical gender language emphasizes gender associations, especially when these two are conceptually bound to each other. Although, our cognition of gender itself may not be fully influenced by grammatical gender, and this is an empirical question, our social cognition may well be, given that the concept of gender, especially that of male, is enhanced in grammatical gender language readers. These tendencies may then result in shifting or influencing our social perceptions of gender-stereotyped occupations, guiding readers to integrate a representation that is advantageous for men (Irmen and Köhncke, 1996; Braun et al., 2005).

## Conclusion

Using a linguistic-visual paradigm, the present study showed that readers automatically activate gender-associated information when reading gender stereotypical human referent role nouns. The activation of such information immediately takes place at a lexical level when readers encounter a role noun. Though morphological markings such as the default masculine form in French and German appear to be central when constructing mental representations of gender rather than superficial surface features, our study demonstrated that stereotype information also plays a role in influencing readers’ mental representations. A stereotype effect was particularly apparent in the cumulative effects of stereotype and grammar when readers encounter male stereotyped role nouns. While past studies had not clearly found the effects of stereotype information in the presence of strong masculine effects (e.g., Gabriel and Gygax, 2008; Garnham et al., 2012; Gygax et al., 2012), the adaptation of a lexical and conceptual paradigm (with visual stimuli) was able to effectively gauge these effects. Future studies may want to further examine the possibilities of suppressing such male-dominant properties, though they appear to be relatively robust.

## Author Contributions

All authors listed, have made substantial, direct and intellectual contribution to the work, and approved it for publication.

## Conflict of Interest Statement

The authors declare that the research was conducted in the absence of any commercial or financial relationships that could be construed as a potential conflict of interest.

The reviewer RHM and handling Editor declared their shared affiliation, and the handling Editor states that the process nevertheless met the standards of a fair and objective review.

## Footnotes

1 Note that grammatical gender does not determine gender for all human references and the relation is mutual. For instance, there are special cases such as bi-gender role nouns where the sex of the referent determines the grammatical gender (e.g., artiste [artist – French] can be either masculine or feminine depending on the gender of the person). Additionally, there are also examples of epicenes where a single gender can refer to both the biological sex (e.g., secrétaire_Feminine_ is always feminine irrespective of whether it refers to a male or a female).

2 As past studies on gender representation (e.g., Gygax and Gabriel, 2008; Gygax et al., 2008; Garnham et al., 2012) did not find effects of participants’ gender in reading tasks, we did not balance the gender sample of our participants.

3 If anything, participants were slightly faster (by 36 ms) to respond to mixed pairs of faces than to male pairs (*p* = 0.08).

4 www.ondaf.de

5 Experiment order for participants’ L1 and L2 was not randomized as Sato et al. (2013) did not find any experimental order effects based on language.
